# Hemokinin-1 Gene Expression Is Upregulated in Microglia Activated by Lipopolysaccharide through NF-κB and p38 MAPK Signaling Pathways

**DOI:** 10.1371/journal.pone.0032268

**Published:** 2012-02-27

**Authors:** Atsushi Sakai, Kumiko Takasu, Makoto Sawada, Hidenori Suzuki

**Affiliations:** 1 Department of Pharmacology, Nippon Medical School, Tokyo, Japan; 2 Department of Brain Function, Research Institute of Environmental Medicine, Nagoya University, Nagoya, Japan; University of Medicine and Dentistry of New Jersey, United States of America

## Abstract

The mammalian tachykinins, substance P (SP) and hemokinin-1 (HK-1), are widely distributed throughout the nervous system and/or peripheral organs, and function as neurotransmitters or chemical modulators by activating their cognate receptor NK_1_. The TAC1 gene encoding SP is highly expressed in the nervous system, while the TAC4 gene encoding HK-1 is uniformly expressed throughout the body, including a variety of peripheral immune cells. Since TAC4 mRNA is also expressed in microglia, the resident immune cells in the central nervous system, HK-1 may be involved in the inflammatory processes mediated by these cells. In the present study, we found that TAC4, rather than TAC1, was the predominant tachykinin gene expressed in primary cultured microglia. TAC4 mRNA expression was upregulated in the microglia upon their activation by lipopolysaccharide, a well-characterized Toll-like receptor 4 agonist, while TAC1 mRNA expression was downregulated. Furthermore, both nuclear factor-κB and p38 mitogen-activated protein kinase intracellular signaling pathways were required for the upregulation of TAC4 mRNA expression, but not for the downregulation of TAC1 mRNA expression. These findings suggest that HK-1, rather than SP, plays dominant roles in the pathological conditions associated with microglial activation, such as neurodegenerative and neuroinflammatory disorders.

## Introduction

Mammalian tachykinins comprise a family of neuropeptides with a common carboxyl terminal amide motif. The representative tachykinin peptide, substance P (SP), is encoded by the TAC1 gene and preferentially activates a G-protein-coupled receptor, NK_1_. The most recently identified tachykinin member, hemokinin-1 (HK-1; encoded by the TAC4 gene), also activates the NK_1_ receptor with similar affinity and potency to SP [1], [2], although the effects of SP and HK-1 are slightly different [3]–[6]. In addition, the TAC4 and TAC1 expression patterns differ considerably. TAC1 is highly expressed in the nervous system, while TAC4 is uniformly expressed throughout the body [7]. TAC4 is expressed in a variety of peripheral immune cells, including lymphocytes, monocytes and macrophages [8]–[10]. Accordingly, HK-1 is involved in immune functions and inflammation [2], [5], [11]–[14]. However, its role in the nervous system remains almost unknown.

Microglia, the resident immune cells in the central nervous system (CNS), also express the TAC4 gene [10], [15], similar to the case for peripheral immune cells. Microglia have significant roles in various neurological diseases, such as neurodegenerative disorders and chronic pain [16], [17]. In these situations, microglia become activated to upregulate and release a variety of inflammatory mediators that contribute to the pathophysiology. Therefore, HK-1 expression in microglia may also be involved in the inflammatory processes mediated by these cells in the CNS. In fact, we previously observed that the TAC4 expression level was increased in the dorsal spinal cord in the neuropathic pain state, in which activated microglia underlie the hyperalgesia. The increase in TAC4 expression was suppressed by inhibiting microglial activation [15]. However, it remains unknown how the tachykinin genes are precisely regulated in microglia.

In this study, we examined the expression levels of the TAC1 and TAC4 tachykinin genes in microglia activated by lipopolysaccharide (LPS), a well-characterized Toll-like receptor 4 (TLR4) agonist. TLR4 is a member of the pattern recognition receptors that mediate innate immunity and induces microglial activation in response to pathogen-derived and endogenous molecules. Upon activation by LPS, TAC4 expression was significantly increased in microglia, while TAC1 expression was decreased. Since TLR4-induced activation of microglia results in the release of inflammatory substances and mediates various CNS diseases through multiple intracellular mechanisms [18], we further examined the signaling pathways responsible for the regulation of tachykinin expressions and found that the increase in TAC4 expression was mediated by nuclear factor (NF)-κB and p38 mitogen-activated protein kinase (MAPK).

## Materials and Methods

### Ethics Statement

All experimental procedures were approved by the Nippon Medical School Animal Care and Use Committee (approval number: 22-054).

### Materials

LPS (from *Escherichia coli*, serotype 0111:B4), Eagle's minimal essential medium and hemokinin peptide were obtained from Sigma-Aldrich (St. Louis, MO). 4-(4-Fluorophenyl)-2-(4-methylsulfinylphenyl)-5-(4-pyridyl)1H-imidazole (SB 203580) and SC-514 were purchased from Merck (Darmstadt, Germany). Recombinant rat interleukin (IL)-1β was from R&D Systems (Minneapolis, MN). A rabbit anti-ionized calcium-binding adaptor molecule 1 (Iba I) antibody was from Wako (Osaka, Japan). Fetal calf serum and an Alexa Fluor 488-conjugated donkey anti-rabbit IgG antibody were from Life Technologies (Carlsbad, CA). VECTASHIELD Mounting Medium with DAPI was from Vector Laboratories (Burlingame, CA).

### Primary microglial culture

Isolated primary microglial cultures were prepared as described previously [19]. Briefly, primary mixed glial cell cultures from whole brains of Sprague-Dawley rats at postnatal day 0–1 were prepared in 75-cm^2^ culture flasks and maintained in Eagle's modified essential medium containing 10% fetal bovine serum, 5 µg/ml of bovine insulin and 2% glucose at 37°C in 5% CO_2_. After 2 weeks, the culture flasks were shaken on an orbital shaker at 120 rpm at 37°C for 2 h and the medium was harvested. Subsequently, microglia were recovered as adherent cells to uncoated plastic dishes (Falcon 1001; Becton-Dickinson Japan, Tokyo, Japan). The adherent microglia were collected with a rubber policeman and plated at a concentration of 5×10^6^ cells per 6-cm plastic dish (Falcon 3002; Becton-Dickinson Japan). The purity of the enriched microglia was more than 97%, as determined by Iba I immunocytochemical staining.

### Drug treatment of microglia

At 1 day after plating, the microglia were treated with LPS (0.1 µg/ml), IL-1β (20 µg/ml), ATP (100 µM) or HK-1 (1 µM) for 24 h. To block the intracellular signaling pathways, the microglia were treated with 30 µM SC-514, an NF-κB activation inhibitor, or 10 µM of SB 203580, a p38 MAPK inhibitor, dissolved in dimethyl sulfoxide at 1 h prior to LPS stimulation. Images of the microglial morphology were captured using a high-resolution digital camera equipped with a computer (Olympus, Tokyo, Japan).

### RNA isolation and quantitative PCR

Quantitative analyses of the rat TAC4, TAC1 and tachykinin receptor (NK_1_, NK_2_ and NK_3_) mRNA levels were performed as previously described [15]. Total RNA was isolated using RNAiso Plus (Takara, Shiga, Japan) according to the manufacturer's recommended protocol. First-strand cDNAs were synthesized using 0.3 µg of total RNA with oligo (dT)_20_ primer and an iScript Select cDNA Synthesis Kit (Bio-Rad Laboratories, Tokyo, Japan). For quantitative PCR, 0.75 µl of the first-strand cDNA was added to 14.25 µl of a reaction mixture containing TaqMan Gene Expression Master Mix, 900 nM of each primer and 250 nM of TaqMan probe and amplified using a Step One Plus Real-Time PCR System (Life Technologies). PCR primers specific for rat TAC4, TAC1, NK_1_, NK_2_, and NK_3_ were designed based on the cDNA sequences deposited in GenBank (AY471575, NM_012666, NM_012667, NM_080768 and NM_017053, respectively) using Primer Express Version 2.0 (Life Technologies). The forward primers, reverse primers and probes were 5′-AGGGCTCGATAAAGGAGTTA-3′, 5′-TTCAGCCCTCTACCCAGCAT-3′ and 5′-TAGGCAGCTTCCTCAGC-3′ for TAC4, 5′-CGCAATGCAGAACTACGAAAGA-3′, 5′-CGCGGACACAGATGGAGAT-3′ and 5′-CGTAAATAAACCCTGTAACGCACTATCTAT-3′ for TAC1, 5′-CACCCGATACCTCCAGACACA-3′, 5′-GGAGCCGTTGGAGGTGAGA-3′ and 5′-CAGCGTGTACAAGGTCAGCCGCCT-3′ for NK_1_, 5′-CTCCACAATGTACAACCCTATCATTTAT-3′, 5′-GAAAGCAAGCCGGAATCCA-3′ and 5′-CTGCCTTAACCACAGGTTTCGCT-3′ for NK_2_ and 5′-CAACTACTGCCGCTTCCAGAA-3′, 5′-GTCCACTGCAATGGCTGTCATA-3′ and 5′-TTCTTTCCCATCACAGCGGTGTTTGC-3′ for NK_3_, respectively. For quantification, the cDNA sequences of TAC4, TAC1, NK_1_, NK_2_ and NK_3_ were inserted into the pBluescript II SK(+) vector (Agilent Technologies, Santa Clara, CA), pcDNA3 vector (Life Technologies) or the pGEM-T easy vector (Promega, Madison, WI). The plasmids were then serially diluted to concentrations of 1.0×10^2^–1.0×10^5^ mol per reaction tube for use as standards. All PCR amplifications using the standards and samples were performed in triplicate at 50°C for 2 min and 95°C for 10 min, followed by 40 cycles of 95°C for 15 s and 60°C for 1 min. The number of cDNA copies was calculated using a standard curve obtained from the set of control plasmids in each assay.

### Immunocytochemical staining of Iba I

Microglia were seeded onto culture slides and allowed to equilibrate for 24 h. The culture medium was removed and the cells were washed in phosphate-buffered saline (PBS; pH 7.2) and fixed with 4% paraformaldehyde for 15 min at room temperature. After two rinses in PBS, the cells were incubated in the presence of 0.2% Triton X-100 for 10 min, rinsed with PBS twice, blocked with 2% donkey serum for 30 min and incubated with a primary rabbit antibody against Iba I (1∶2000 dilution) at 4°C overnight. After three washes in PBS, the cells were incubated with an anti-rabbit IgG antibody conjugated with Alexa Fluor 488 (1∶1000 dilution) at room temperature for 1 h. To identify the stained and unstained cells, the slides were mounted in VECTASHIELD Mounting Medium with DAPI, and images were captured using a high-resolution digital camera equipped with a computer (Olympus).

### Statistical analysis

Values are presented as means ± SEM. Differences in the mRNA expression levels were analyzed by an unpaired *t*-test or one-way repeated-measures ANOVA followed by individual post hoc multiple comparisons (Dunnett's test or Tukey–Kramer multiple comparison test). Values of *P*<0.05 were considered to indicate statistical significance.

## Results

### Changes in TAC4, TAC1 and tachykinin receptor mRNA expressions in microglia following LPS treatment

**Figure 1:**
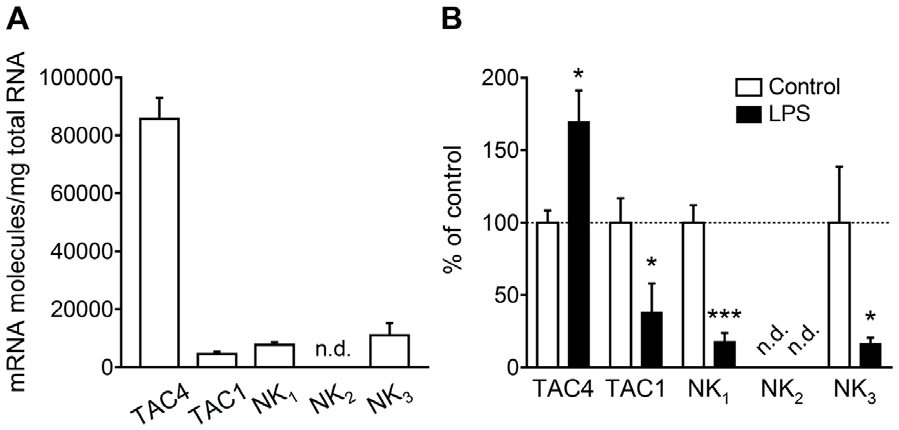
Tachykinin gene expressions in microglia activated by LPS. (A) Numbers of mRNA molecules in non-stimulated microglia. (B) Expression changes in mRNAs in microglia stimulated with LPS (0.1 mg/ml) for 24 h. Data are expressed as percentages of the control microglia. n.d., not detected. *P<0.05 and ***P<0.001, compared with the value in non-stimulated control microglia by an unpaired *t*-test (*n* = 5–9).

In non-stimulated control microglia, the numbers of TAC4 and TAC1 mRNA molecules were 8.6×10^4^±7.2×10^3^ (*n* = 5) and 4.5×10^3^±7.6×10^2^ (*n* = 7) molecules/µg total RNA, respectively (Fig. 1A). In addition, the mRNA expression levels of NK_1_, as the receptor for HK-1 and SP, and NK_3_ were 7.7×10^3^±9.2×10^2^ and 1.1×10^4^±4.2×10^3^ mol/µg total RNA, while NK_2_ mRNA expression was not detected (*n* = 6–7; Fig. 1A). In the microglia stimulated with LPS for 24 h, the expression level of TAC4 mRNA was increased compared with the level in non-stimulated control microglia (169.5±21.7% expressed as a percentage of the control; *P*<0.05; *n* = 5–7; Fig. 1B). In contrast, the expression levels of TAC1, NK_1_ and NK_3_ mRNAs were significantly decreased to 38.2±19.8%, 17.9±5.9% and 16.5±4.0% of the corresponding levels in non-stimulated microglia (*P*<0.05 for TAC1, *P*<0.001 for NK_1_ and *P*<0.05 for NK_3_; *n* = 6–9; Fig. 1B).

### No changes in TAC4 mRNA expression in microglia following IL-1β, ATP or HK-1 treatment

**Figure 2:**
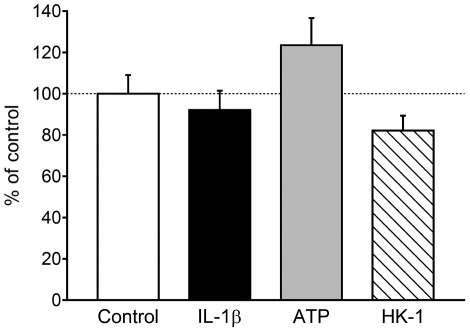
TAC4 expression in microglia stimulated by IL-1β, ATP and HK-1. Expression levels of TAC4 in microglia stimulated with IL-1β (20 mg/ml), ATP (100 µM) or HK-1 (1 µM) for 24 h. Data are expressed as percentages of the control microglia (*n* = 4–9).

In addition to LPS, other molecules such as IL-1β and ATP can also activate microglia [20]. Therefore, we examined the effects of IL-1β and ATP on the TAC4 mRNA expression. However, treatment of the microglia with IL-1β or ATP for 24 h did not significantly increase the TAC4 mRNA expression levels (92.1±9.3% and 123.5±13.1% expressed as percentages of the control, respectively; *n* = 4–9; Fig. 2). Next, we examined whether the upregulation of TAC4 mRNA expression was induced in autocrine and/or paracrine manners because microglia expressed NK_1_ receptors of their own (Fig. 1A). However, HK-1 treatment for 24 h had no effect on the cell number and the expression level of TAC4 mRNA (82.1±7.3% expressed as a percentage of the control; *n* = 7–9; Fig. 2).

### NF-κB and p38 MAPK inhibitors attenuate the LPS-induced increase in the expression level of TAC4 mRNA

**Figure 3:**
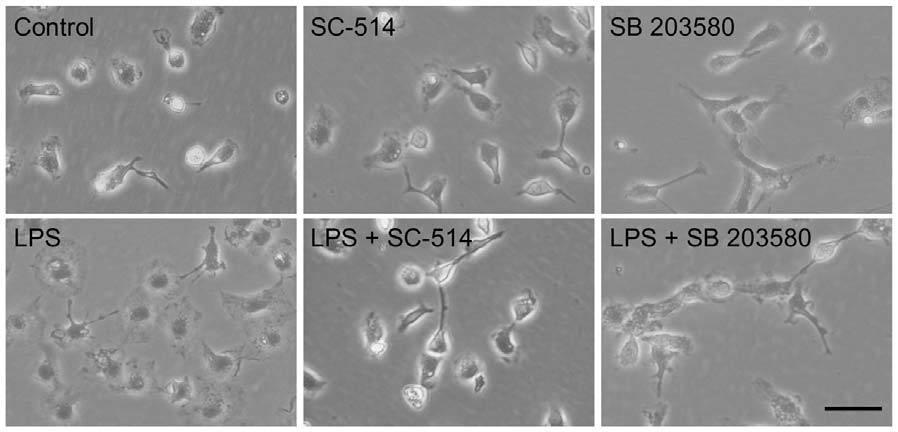
Morphological changes of microglia activated by LPS. Representative images of the microglial morphology are shown. Scale bar, 50 µm.

The TLR4 activation induced by LPS is mainly mediated by NF-κB and MAPK pathways [21]. In addition, we previously demonstrated that HK-1 gene expression is increased in the dorsal spinal cord after peripheral nerve injury and blocked by a microglial inhibitor [15]. Among the MAPKs, p38 MAPK is consistently activated by injury in microglia [22]. Therefore, we pretreated microglia with SC-514 and SB 203580 to block the activations of NF-κB and p38 MAPK, respectively. The morphology of non-stimulated microglia was characterized by bipolar or unipolar processes and elongated cell bodies, while microglia activated by LPS exhibited amoeboid cell bodies with retraction of extensions (Fig. 3). In addition, LPS increased the number of microglia. However, the morphological and numerical changes induced by LPS treatment were greatly attenuated in the microglia pretreated with SC-514 or SB 203580 (Fig. 3). The microglial morphology and cell number were unaffected by SC-514 or SB 203580 alone.

**Figure 4:**
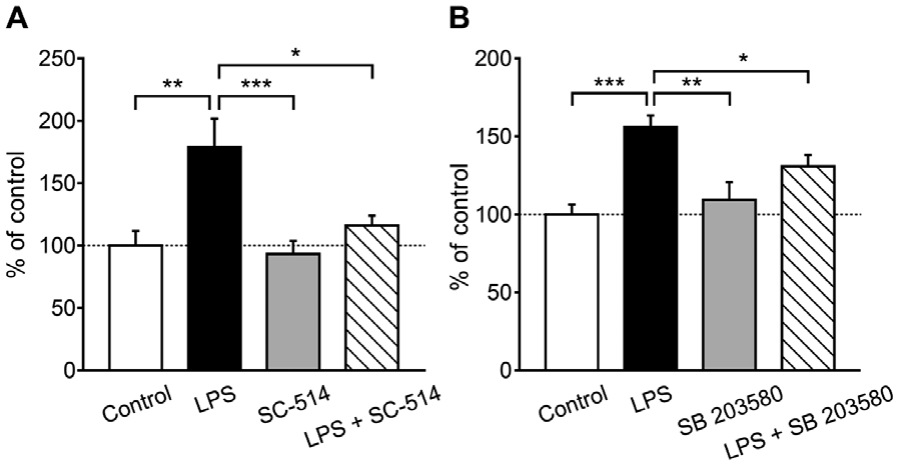
Intracellular signaling pathways for TAC4 gene upregulation by LPS. Expression levels of TAC4 mRNA in microglia treated with LPS plus SC-514 (A; an IKK-2 inhibitor) or SB 203580 (B; a p38 MAPK inhibitor). *P<0.05, **P<0.01 and ***P<0.001 by Turkey-Kramer's multiple comparison test (*n* = 6–10).

Next, we investigated the involvement of the NF-κB and p38 MAPK pathways in the upregulation of TAC4 mRNA expression in the LPS-treated microglia. SC-514 significantly inhibited the upregulation of TAC4 mRNA expression in the LPS-treated microglia (179.0±22.8% for LPS alone and 115.9±8.0% for LPS plus SC-514 expressed as percentages of the control; *P*<0.05; *n* = 6–9; Fig. 4A). SB 203580 also significantly attenuated the upregulation of TAC4 mRNA expression (156.0±7.3% for LPS alone and 130.8±7.2% for LPS plus SB 203580 expressed as percentages of the control; *P*<0.05; *n* = 8–10; Fig. 4B). SC-514 or SB 203580 treatment alone had no effect on the expression levels of TAC4 mRNA (109.3±11.4% for SB 203580 and 93.1±10.5% for SC-514 expressed as percentages of the control; *n* = 8; Fig. 4). Pretreatment with dimethyl sulfoxide did not affect the upregulation of TAC4 mRNA expression induced by LPS (94.9±23.6% expressed as a percentage of the value for LPS alone; *n* = 4).

### NF-κB and p38 MAPK inhibitors are not involved in the LPS-induced decrease in the expression levels of TAC1, NK_1_ and NK_3_ mRNAs

**Figure 5:**
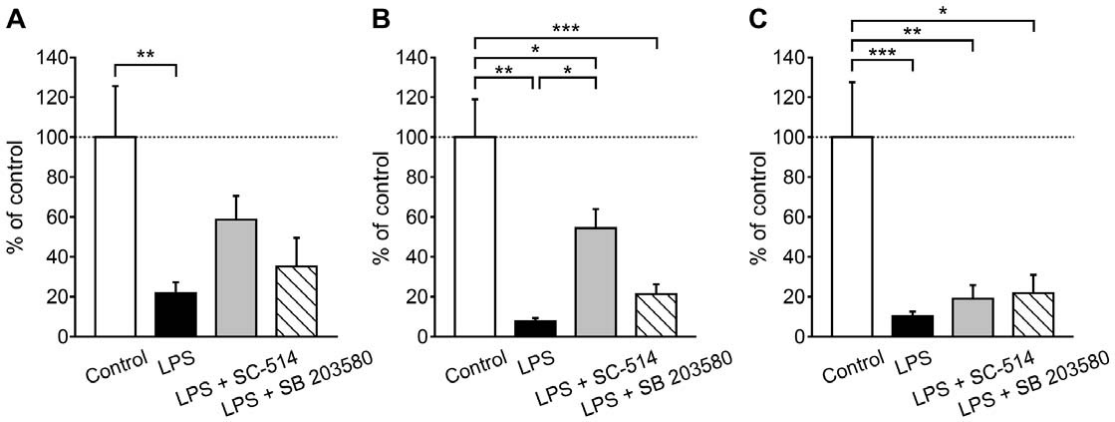
Intracellular signaling pathways for TAC1, NK_1_ and NK_3_ gene downregulation by LPS. Expression levels of TAC1 (A), NK_1_ (B) and NK_3_ (C) mRNAs in microglia activated by LPS plus SC-514 or SB 203580. *P<0.05, **P<0.01 and ***P<0.001 by Turkey-Kramer's multiple comparison test (*n* = 6–14).

We further examined the involvement of NF-κB and p38 MAPK in the downregulation of TAC1, NK_1_ and NK_3_ mRNA expressions. Neither SB 203580 nor SC-514 significantly inhibited the downregulation of TAC1 and NK_3_ mRNA expressions in the LPS-treated microglia (21.9±5.4% and 10.3±2.3% for LPS alone, 35.2±14.3% and 21.8±9.1% for LPS plus SB 203580 and 58.6±11.9% and 19.1±6.7% for LPS plus SC-514 expressed as percentages of the control, respectively; *n* = 8–14; Fig. 5A), although SC-514 showed a tendency to partially inhibit the downregulation of TAC1 mRNA expression. The decreases in the NK_1_ mRNA expression levels were also unaffected by SB 203580 (7.8±1.6% for LPS alone and 21.3±4.9% for LPS plus SB 203580 expressed as percentages of the control; *n* = 6–8; Fig. 5B). On the other hand, SC-514 partially, but significantly, inhibited the downregulation of NK_1_ mRNA expression (54.3±9.6% for LPS plus SC-514 expressed as a percentage of the control; *P*<0.05; *n* = 6–8; Fig. 5B).

## Discussion

In this study, we found that TAC4, rather than TAC1, was the predominant tachykinin gene expressed in microglia. Furthermore, the TAC4 mRNA expression was upregulated in microglia upon their activation by LPS, while the TAC1 mRNA expression was downregulated. Both the NF-κB and p38 MAPK signaling pathways were required for the upregulation of TAC4 mRNA, but not for the downregulation of TAC1 mRNA.

### HK-1 is the predominant tachykinin in activated microglia

In the non-stimulated condition, TAC4 mRNA was expressed in primary cultures of microglia, as previously described for microglial cell lines [10], [15]. The expression level was much higher than that of TAC1 mRNA. In addition, the expression of TAC4 mRNA was increased in activated microglia, while TAC1 mRNA expression was decreased. These findings suggest that HK-1 is the predominant endogenous NK_1_ receptor ligand expressed in microglia, and that SP and HK-1 are expressed in distinct CNS cells. In fact, SP is preferentially expressed in neurons [23] and its mRNA expression level is much higher than that of TAC4 mRNA expression in the nervous system [7]. On the other hand, TAC4 mRNA is expressed in the resident immunocompetent microglia, similar to the peripheral tissues where a variety of peripheral immune cells, including macrophages and dendritic cells, express TAC4 mRNA [5], [8]–[10]. TAC4 mRNA expression is uniform throughout the whole body, and the intact brain also expresses TAC4 mRNA at comparable amounts to peripheral tissues [7]. In addition, many reports have intriguingly indicated differences in the effects of SP and HK-1 [3]–[6], although both peptides activate NK_1_ receptors with comparable high affinity and potency and both NK_2_ and NK_3_ receptors with lower affinities [1], [2]. Therefore, HK-1 may be distinct from SP not only in its expressing cells, but also in its roles in the CNS.

TAC4 mRNA expression was upregulated in microglia activated by the TLR4 agonist LPS, suggesting that HK-1 may mediate the microglial actions on neighboring cells, such as neurons and astrocytes. Although LPS is a component of gram-negative bacterial cell walls, TLR4 was also reported to be activated by various endogenous molecules, including heat shock proteins and high mobility group box 1 protein [18], [24]. We previously showed that the expression of TAC4 mRNA was upregulated by peripheral nerve injury in association with microglial activation in the dorsal spinal cord. A microglial activation inhibitor, minocycline, suppressed both the upregulation of HK-1 mRNA and neuropathic pain caused by the injury [15]. Neuropathic pain and microglial activation are also attenuated in TLR4-deficient mice [25]. Intrathecal administration of HK-1 induces nociceptive behavior [6], [26]. On the other hand, HK-1 was reported to potentiate the antinociceptive effects of peripheral and supraspinal opioids [27], [28]. These findings suggest that an increase in HK-1 expression can actually be induced to mediate the microglial actions in the CNS.

### Intracellular signaling pathway for the modulation of HK-1 gene expression

The upregulation of HK-1 gene expression in the microglia activated by LPS required NF-κB and p38 MAPK signaling pathways. NF-κB was shown to be involved in the increase in TAC4 gene transcription by phorbol myristate acetate in a T cell line [29], consistent with the present data showing that NF-κB was prerequisite for LPS-induced HK-1 mRNA upregulation in microglia. In addition, we found that p38 MAPK, which is involved in the transcriptional regulation of a variety of inflammatory cytokines [30], was also required for TAC4 mRNA upregulation. Concomitant inhibition of NF-κB and p38 MAPK may exert synergistic inhibition as in the case of cytokine expression [21]. However, IL-1β, which also induces inflammatory mediators in microglia, did not induce HK-1 gene expression, although both LPS and IL-1β lead to the activation of NF-κB and p38 MAPK [31]. ATP was reported to induce p38 MAPK activation in microglia [20], [32]. These findings suggest that LPS-induced TAC4 expression requires other additional signaling molecules that are not recruited by IL-1β. The LPS/TLR4 signaling pathways are composed of myeloid differentiation factor 88 (MyD88)-dependent and -independent pathways [21], while IL-1β functions through the MyD88-dependent pathway [33]. Therefore, the MyD88-independent pathway, which is unique to TLR3 and TLR4, may also be required for the upregulation of TAC4 mRNA expression and downregulation of TAC1 and NK receptors. NF-κB modulates the transcription of target genes, including type I interferons, in a cooperative manner with interferon regulatory factor-3, a transcription factor activated through an MyD88-independent pathway [21], [34].

The modulation of HK-1 expression also seems to depend on the cell types. In a pre-B cell line and dendritic cells, TAC4 mRNA expression was reported to be decreased by LPS [5], [10]. Although the reason for the discrepancy remains unknown, TLR4 activation reportedly induces different gene expressions via similar intracellular signaling pathways among distinct cell types, such as dendritic cells, microglia and neurons [35]. The expression of TAC4 mRNA in human B lymphocytes is upregulated in chronic lymphocytic leukemia and non-Hodgkin's lymphoma, but downregulated in acute lymphoblastic leukemia [36]. In monocyte and macrophage cell lines, both HK-1 and NK_1_ gene expressions are downregulated after co-stimulation with interferon-γ, IL-1β and tumor necrosis factor-α [8].

### Pathophysiological relevance of HK-1 in microglia

Activated microglia play important roles in neurodegenerative disorders and neuroinflammation, including Alzheimer's disease, Parkinson's disease and amyotrophic lateral sclerosis [16], [18]. TLR-induced activation of microglia is responsible for neurotoxic processes in these diseases [18]. On the other hand, microglia also exert neuroprotective effects in situations such as ischemic lesions [37]. The present data indicate that HK-1, rather than SP, may play dominant roles in the pathological and/or restorative situations associated with microglial activation. Therefore, further investigation of the roles of HK-1 in connection with microglial activation may lead to novel insights into the pathophysiology of neurodegenerative and neuroinflammatory disorders.
